# Development and Application of Indirect ELISA for IBDV VP2 Antibodies Detection in Poultry

**DOI:** 10.3390/v17070871

**Published:** 2025-06-20

**Authors:** Wenying Zhang, Yulong Wang, Guodong Wang, Hangbo Yu, Mengmeng Huang, Yulong Zhang, Runhang Liu, Suyan Wang, Hongyu Cui, Yanping Zhang, Yuntong Chen, Yulong Gao, Xiaole Qi

**Affiliations:** 1 Avian Immunosuppressive Diseases Division, State Key Laboratory for Animal Disease Control and Prevention, Harbin Veterinary Research Institute, The Chinese Academy of Agricultural Sciences, Harbin 150069, China; zhangwenying0402@163.com (W.Z.); xyylong@126.com (Y.W.); setback1231@163.com (G.W.); yuhangbo2022@163.com (H.Y.); huangmm1017@163.com (M.H.); yulozh@163.com (Y.Z.); lrh18730280216@163.com (R.L.); wangsuyan@caas.cn (S.W.); cuihongyu@caas.cn (H.C.); zhangyanping@caas.cn (Y.Z.); chenyuntong@caas.cn (Y.C.); 2 World Organization for Animal Health (WOAH) Reference Laboratory for Infectious Bursal Disease, Harbin Veterinary Research Institute, The Chinese Academy of Agricultural Sciences, Harbin 150069, China; 3 Jiangsu Co-Innovation Center for the Prevention and Control of Important Animal Infectious Disease and Zoonosis, Yangzhou University, Yangzhou 225009, China

**Keywords:** IBDV, VP2, VLP, ELISA, antibody detection

## Abstract

Infectious bursal disease virus (IBDV) is one of the most important immunosuppressive viruses in poultry, causing the global spread of infectious bursal disease (IBD). It poses a significant threat to the healthy development of the poultry industry. Vaccination is an effective approach for controlling IBDV infection. Therefore, reliable immune monitoring for IBDV is critical for maintaining poultry health. The enzyme-linked immunosorbent assay (ELISA) is a common technique used to detect specific antibodies in clinical serum testing and for the serological evaluation of IBDV vaccines. Among the currently available and under development IBDV vaccines, IBD VP2 subunit-based vaccines account for a considerable proportion. These vaccines stimulate the production of antibodies that are specific only to VP2. However, most IBDV antibody ELISA kits approved for use have applied the whole virus as the coating antigen, which does not adequately meet the diverse requirements for IBDV detection across different conditions. This study utilized a prokaryotic expression system to express the VP2 protein of the IBDV epidemic strain, assembling it into virus-like particles to be used as coating antigens. This approach enabled the establishment of an indirect ELISA method for detecting IBDV VP2 antibody (VP2-ELISA). The optimal coated antigen concentration was determined to be 2.5 μg/mL, with overnight coating at 4 °C; sealing with 5% skim milk at 37 °C for 4 h; serum dilution at 1:500 with incubation at 37 °C for 30 min; secondary antibody dilution at 1:4000 with incubation at 37 °C for 40 min; and then incubation with the substrate solution 3,3′,5,5′-tetramethylbenzidine at room temperature for 20 min. The criterion for interpreting the detection results was OD_450nm_ ≥ 0.111 indicates IBDV antibody positivity, while OD_450nm_ < 0.111 indicates negativity. The established VP2-ELISA can specifically detect IBDV-positive sera at the lowest serum dilution of 1:6400, with intra- and inter-batch coefficients of variation of <2%. This indicates that the VP2-ELISA exhibits good specificity, sensitivity, and stability. Detection experiments using 20 laboratory-immunized chicken serum samples and 273 clinical serum samples demonstrated that the results of VP2-ELISA were consistent with those of commercial ELISA kits coated with whole virus. In summary, the VP2-ELISA developed in this study offers advantages in immune response detection for IBD VP2 subunit-based vaccines and is appropriate for evaluating the efficacy of IBD vaccines and detecting clinical serum samples.

## 1. Introduction

Infectious bursal disease virus (IBDV), one of the most immunosuppressive viruses, belongs to the genus *Avibirnavirus* within the family *Birnaviridae* [1,2]. The viral genome consists of two segments of double-stranded RNA—segment A (3.2 kb) and segment B (2.8 kb). Segment A contains two overlapping open reading frames (ORFs) that encode two proteins—polyprotein (NH3-VP2-VP4-VP3-COOH) and the non-structural protein VP5. Polyprotein is self-cleaved into the precursors VP2 (pVP2), VP3, and VP4 [3]. During virus replication and capsid assembly, pVP2 is further processed into mature VP2 [4,5,6,7]. VP2 is the capsid protein of IBDV [8], capable of stimulating the host immune system, generating neutralizing antibodies, and serving as the main protective antigen of IBDV [9,10,11,12]. Accordingly, VP2 is the most critical target for developing IBDV vaccines.

The currently available and under development IBDV vaccines can generally be divided into two main categories based on their antigenic components [13]. The first category includes whole virus vaccines, such as attenuated live vaccines, intermediate live vaccines, inactivated vaccines, and antigen-antibody complex vaccines. The second category comprises IBD VP2 subunit-based vaccines, wherein the antigenic component is the VP2 protein of IBDV. Examples include subunit vaccines [14], live viral vector vaccines expressing IBDV VP2, and multivalent vaccines containing IBDV VP2 subunits [15,16]. Currently, IBD subunit-based vaccines account for a considerable proportion of the available vaccines. Enzyme-linked immunosorbent assays (ELISAs) are widely used in clinical detection and vaccine evaluation owing to their high-throughput capabilities. However, the coating antigen of IBDV antibody ELISA kits approved for use in various countries, including China, is the whole virus of IBDV, which does not adequately meet the diverse requirements for IBDV detection across varying conditions. Studies have shown that directly detecting VP2 antibodies, rather than whole virus antibodies, is more accurate and practical for evaluating the immune effects of these vaccines [17]. Additionally, compared to whole virus preparation, VP2 protein preparation is simpler, more cost-effective, and safer. Therefore, establishing an indirect ELISA detection method for IBDV VP2 antibodies using VP2 protein as the coating antigen is of considerable value for vaccine evaluation and the comprehensive prevention and control of IBD.

This study utilized a prokaryotic expression system to express the VP2 protein of the IBDV epidemic strain and prepare it into virus-like particles (VLPs). These VLPs were used as coating antigens to establish an indirect ELISA method for detecting IBDV VP2 (referred to as VP2-ELISA in this study), providing a new technology for the detection and prevention of IBDV.

## 2. Materials and Methods

### 2.1. Plasmids, Bacterial Strains, Antibodies, and Sera

The recombinant prokaryotic expression vector pCo-HHT28-SHG19VP2-466, which expresses the VP2 protein of the IBDV representative strain SHG19 (GenBank accession No. MN393076), was prepared by the Avian Immunosuppressive Disease Division, Harbin Veterinary Research Institute, Chinese Academy of Agricultural Sciences (hereinafter referred to as “our lab”). The pCo-HHT28-SHG19VP2-466 vector was transformed into the engineered bacterium *E. coli* BL21 (DE3) (Takara, Beijing, China), and the resulting recombinant prokaryotic expression bacterium was named IBDV-VLP-DE3 [14]. The monoclonal antibody (MAb) 7D4 against IBDV VP2 was prepared and stored in our lab. The anti-His antibody was obtained from Biodragon (Suzhou, China). The IBDV-positive serum and IBDV-negative serum were prepared and stored in our lab. Positive sera for Marek’s disease virus (MDV), avian metapneumovirus (aMPV), chicken anemia virus (CAV), fowl adenovirus (FAdV), and avian leukosis virus subtypes A (ALV-A), B (ALV-B), and J (ALV-J) were also prepared and stored in our lab. Positive sera for the H5, H7, and H9 subtypes of avian influenza virus (AIV) were purchased from Harbin Weike Biotechnology Co., Ltd. (Harbin, China).

### 2.2. Expression and Purification of IBDV-VLPs

Recombinant VP2 protein (IBDV-VLP) was prepared as described in our previous publication [14]. The frozen recombinant bacterial strain IBDV-VLP-DE3 was inoculated into LB medium at a volume ratio of 1% and cultured at 37 °C with shaking at 220 rpm for 12 h. The revitalized IBDV-VLP-DE3 culture was mixed with LB at a 1:100 ratio (*v*/*v*) and incubated at 37 °C with shaking at 220 rpm for 2–3 h. The optical density at 600 nm (OD_600nm)_ was measured using a spectrophotometer. When the OD_600nm_ reached 0.6, the culture was cooled on ice for 10 min. IPTG was added to induce protein expression for 20 h. After induction, the culture was centrifuged at 8000× *g* for 10 min at room temperature. The supernatant was discarded, and the cell pellets were resuspended in PB 7.5 solution. The cells were sonicated for 40 min and centrifuged again at 8000× *g* and 4 °C for 10 min. The supernatant was collected, and the pellets were discarded. The protein solution was brought to room temperature and saturated with (NH_4_)_2_SO_4_ by slowly adding it to the protein solution until a final concentration of 50% was achieved. After stirring for 3 min, the solution was centrifuged at 8000× *g* and 4 °C for 10 min. The supernatant was discarded, and the pellets were thoroughly resuspended in a PB 7.5 solution. The suspension was centrifuged at 8000× *g* and 4 °C for 10 min. After centrifugation, the supernatant was filtered through a 0.45 μm filter. The sample was then purified using Tangential Flow Filtration (TFF) until the protein solution was concentrated to 50 mL. Finally, the sample was collected after three washing steps.

### 2.3. Identification of IBDV-VLP

SDS-PAGE and Western blotting: The IBDV-VLP samples formed by VP2 protein were subjected to sodium dodecyl sulfate–polyacrylamide gel electrophoresis (SDS-PAGE, followed by Western blot analysis to confirm the expression of the IBDV VP2 protein, with a target protein size of approximately 55 kDa. The antibody used for Western blotting was MAb 7D4 against IBDV VP2. The secondary antibody was IRDye 800CW goat anti-mouse IgG (Li-Cor Biosciences, Lincoln, NE, USA). Both primary and secondary antibodies were diluted at a ratio of 1:5000 in PBS containing 5% BSA.

Transmission electron microscopy (TEM): Negative staining electron microscopy was performed to observe the assembly of the IBDV VP2 protein into VLPs.

Quantification of protein concentration: The protein concentration of the target protein was determined using a BCA Protein Quantification Kit (Thermo, Waltham, MA, USA). Serially diluted protein standards were separated on SDS-PAGE along with the samples. After gel scanning, the band intensity of the samples and standards was measured using ImageJ software (Version 1.8.0). The protein concentration of the samples was calculated from a linear equation derived from the standard curve.

### 2.4. Development and Optimization of VP2-ELISA

Checkerboard titration was performed to determine the optimal amount of coating antigen and the optimal dilution of chicken serum samples in each well. The IBDV-VLP antigen was diluted from 10 μg/mL to 1.25 μg/mL (two-fold dilution). Ninety-six-well polystyrene microtiter plates (Costar, New York, NY, USA) were coated overnight at 4 °C with purified IBDV-VLPs diluted in carbonate buffer (pH 9.6). Following incubation with the coating antigen, the plates were washed five times with PBS containing 0.05% Tween 20 and then blocked with 5% skim milk in phosphate-buffered saline (PBS) containing 0.05% Tween 20 (pH 7.4). Blocking was performed in 100 μL for 1, 2, 3, or 4 h at 37 °C. After five washes, the IBDV-positive and IBDV-negative serum samples were diluted from 1:250 to 1:1000 (two-fold dilution) and incubated at 37 °C for 30, 45, or 60 min. Subsequently, the plates were washed five times and incubated at 37 °C for 20, 40, or 60 min with HRP-conjugated rabbit anti-chicken antibodies (Sigma, Livonia, MI, USA) diluted at 1:3000, 1:4000, or 1:5000. After washing, 100 μL of 3,3′,5,5′-tetramethylbenzidine (TMB) substrate (Amresco, Solon, OH, USA) was added and incubated for 5, 10, 15, or 20 min in the dark. The enzymatic reaction was quenched using sulfuric acid and the optical density (OD) was recorded at 450 nm. The ODs represent the mean values of three replicate wells. The ratio of the optical density at 450 nm (OD_450nm_) of the positive serum sample (P) to that of the negative serum sample (N), known as the P/N value, was maximized under optimal reaction conditions. After determining the optimal conditions for antigen coating and serum sample dilution, the optimal times for well blocking, serum incubation, antibody incubation, and TMB incubation were then determined.

### 2.5. Determination of the Cut-Off Value

A total of 128 negative serum samples were collected. The OD_450nm_ values of these serum samples, obtained via VP2-ELISA, were recorded to calculate the cut-off value. The cut-off value was determined using the following formula: Cut-off value = mean OD_450nm_ of negative samples (N) + 3 × Standard Deviations (SDs).

### 2.6. Specificity Assay

The specificity of the VP2-ELISA established in this study was determined by comparing the OD_450nm_ values of the positive serum samples for ALV-A, ALV-B, ALV-J, AIV-H5, AIV-H7, AIV-H9, CAV, FADV, MDV, and aMPV measured using the VP2-ELISA and a commercial IBDV antibody ELISA kit. The serum samples were diluted 1:500, and three replicates of each sample were tested simultaneously. The OD_450nm_ values for each sample were calculated.

### 2.7. Sensitivity Assay

The sensitivity of the VP2-ELISA established in this study was determined using serial dilutions of IBDV-positive serum samples (1:100, 1:200, 1:400, 1:800, 1:1600, 1:3200, 1:6400, and 1:12,800). Comparative tests were performed using a commercial IBDV-ELISA and an indirect immunofluorescence assay (IFA). According to the instructions of this commercial IBDV-ELISA kit, the antibody titer (875) was defined as the criterion for interpreting the test results: titer > 875, IBDV antibody positive. For IFA, DF1 cells were infected with IBDV for 24 h, then the DF1 cells were fixed with ice-cold 4% paraformaldehyde for 15 min. After washing with PBST, the cells were blocked with 5% skim milk at 37 °C for 30 min. The IBDV positive serum was used as the primary antibody at a 1:100 to 1:6400 dilution in PBS, and incubation was performed at 37 °C for 1 h, followed by washing with PBST. Anti-mouse IgG-FITC (Sigma–Aldrich, Livonia, MI, USA) was added and incubated for 45 min, followed by washing three times with PBST. SPF chicken serum was used as the negative control (NC). An inverted fluorescence microscope was used to observe the results.

### 2.8. Repeatability Assay

To evaluate the repeatability of the developed VP2-ELISA, plates were prepared using the same batch (intra-batch) and different batches (inter-batch) of purified IBDV-VLPs as the coating antigen. IBDV-positive serum samples were selected for indirect VP2-ELISA detection, with each serum sample tested in triplicate. The OD_450nm_ values were recorded, and the SDs and means of the samples were calculated. The coefficient of variation (CV) was calculated using the following formula: CV = (SD/Mean) × 100%. This formula was used to evaluate variability within and between batches.

### 2.9. Detection of Laboratory Samples

To assess the practicality of the VP2-ELISA in evaluating vaccine immune responses, two representative IBD vaccines were selected for laboratory immunization experiments.

#### 2.9.1. IBD Live Vaccine Immunization Experiment and Serum Sample Detection

The commercial live IBD vaccine (Gt), which is a whole-virus vaccine for IBD, was used. According to the vaccine instructions, ten 11-day-old SPF chickens were immunized with the vaccine via the ocular and nasal routes, with one dose per chicken. Fourteen days post-immunization (d.p.i.), serum samples were collected and tested for antibodies using the VP2-ELISA established in this study and a commercial IBDV-ELISA. Serum samples with inconsistent results between the two methods were verified using IFA.

#### 2.9.2. Avian Quadrivalent Vaccine Immunization Experiment and Serum Sample Detection

The commercial avian quadrivalent vaccine (ND-IB-AI-IBD), which uses the IBDV VP2 protein as an IBD antigen component, represents a VP2 subunit-based vaccine for IBD. According to the vaccine instructions, ten 11-day-old SPF chickens were immunized with the quadrivalent vaccine via the ocular and nasal routes, with one dose per chicken. At 14 dpi, serum samples were collected and tested as described above.

### 2.10. Detection of Clinical Samples

A total of 273 clinical chicken serum samples from farms suspected of having IBD cases were tested using the VP2-ELISA established in this study and the commercial IBDV-ELISA.

### 2.11. Statistical Analysis

Graphpad Prism software (version 9.0; GraphPad Software, San Diego, CA, USA) was employed for the analysis of all data.

## 3. Results

### 3.1. Preparation and Identification of IBDV-VLPs

**Figure 1:**
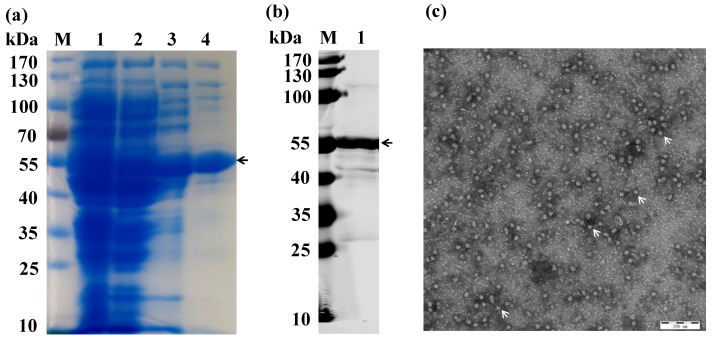
Expression and purification of IBDV-VLP. (**a**) SDS-PAGE analysis of IBDV-VLP; M: 170 kDa protein marker; Lane 1: The original solution of IBDV-VLP; Lane 2: The product after extracting the endotoxin; Lane 3: The purification product of IBDV-VLP using ammonium sulfate precipitation; Lane 4: The purification product of IBDV-VLP using tangential flow filtration. (**b**) Western blotting of IBDV-VLP using IBDV VP2 MAb; M: 170 kDa protein marker; Lane 1: IBDV-VLP. (**c**) TEM image of IBDV-VLP. The VLPs are highlighted with arrows.

Recombinant *E. coli* IBDV-VLP-DE3 was revitalized and subjected to induced expression. SDS-PAGE analysis showed successful expression and purification of the VP2 protein (55 kDa) following ammonium sulfate saturation and TFF (Figure 1a). Western blot analysis confirmed that the expressed VP2 was recognized by the IBDV VP2 MAb (Figure 1b). TEM revealed that the expressed VP2 assembled into VLPs (IBDV-VLPs), with a diameter of approximately 25 nm (Figure 1c). The concentration of the purified IBDV-VLPs was determined to be 500 μg/mL.

### 3.2. Development and Optimization of VP2-ELISA

The optimization process included adjustments to the antigen coating concentration, blocking time, serum dilution, incubation duration, secondary antibody incubation time, and chromogenic time. The following optimal reaction parameters were established: the antigen coating concentration was 2.5 µg/mL, with overnight coating at 4 °C; blocking was performed with 5% skim milk at 37 °C for 4 h; serum was diluted at 1:500 and incubated at 37 °C for 30 min; the secondary antibody was diluted at 1:4000 and incubated at 37 °C for 40 min; and then the substrate solution TMB was incubated at room temperature for 20 min. All experiments were performed in triplicate. Using 128 negative serum samples, the cut-off value was calculated based on the mean OD and SD values, which were 0.069 and 0.014, respectively. Therefore, the detection criterion was defined as follows: OD_450nm_ ≥ 0.111: IBDV antibody positive, OD_450nm_ < 0.111: IBDV antibody negative.

### 3.3. Evaluation of VP2-ELISA

**Figure 2:**
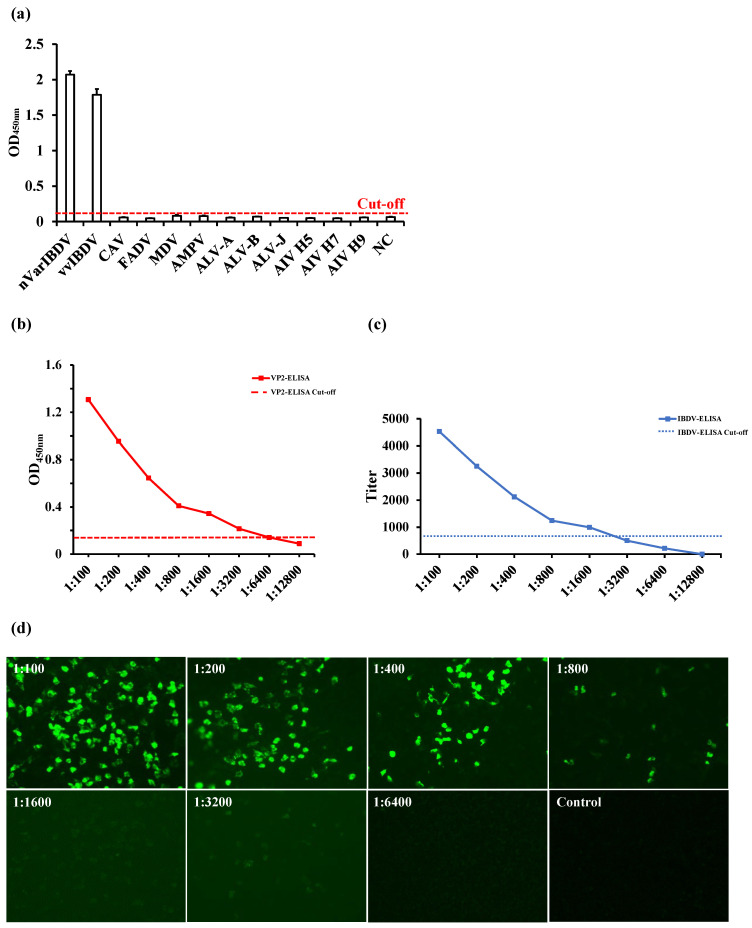
Evaluation of the specificity and sensitivity of VP2-ELISA. (**a**) Specificity detection of two IBDV positive serum samples and ten other pathogen positive serum samples by VP2-ELISA. (**b**,**c**) Comparison of sensitivity between VP2-ELISA (**b**) and IBDV-ELISA (**c**) for detecting the same IBDV positive serum. The cut-off values of VP2-ELISA (OD_450_, 0.111) and IBDV-ELISA (antibody titer, 875) are marked. (**d**) The sensitivity of IFA for detecting the same IBDV positive serum used in (**b**,**c**).

The OD_450nm_ values of the positive sera from common avian pathogens, including CAV, FADV, MDV, aMPV, ALV-A, ALV-B, ALV-J, AIV-H5, AIV-H7, and AIV-H9, were all found to be below 0.111 when tested using VP2-ELISA, indicating negative results. In contrast, sera positive for IBDV had OD_450nm_ values greater than 0.111 (the cut-off value), indicating positive results. These data demonstrated that the VP2-ELISA possessed high specificity (Figure 2a). The sensitivity of the VP2-ELISA was determined and compared with that of the commercial IBDV-ELISA. The lowest detectable serum dilution using the VP2-ELISA was 1:6400 (Figure 2b), while the minimum detectable serum dilution of the commercial IBDV ELISA kit coated with the whole virus antigen was 1:1600 (Figure 2c). In comparison, the detection sensitivity of IFA for the same serum was 1:3200 (Figure 2d). The intra- and inter-batch CV were below 2% (Table 1), indicating that the VP2-ELISA exhibited excellent specificity, sensitivity, and reproducibility.

### 3.4. Detection of Serum Samples from Laboratory Immunized Chickens

#### 3.4.1. Detection of Serum Samples from Chickens Immunized with IBD Live Vaccine

**Figure 3:**
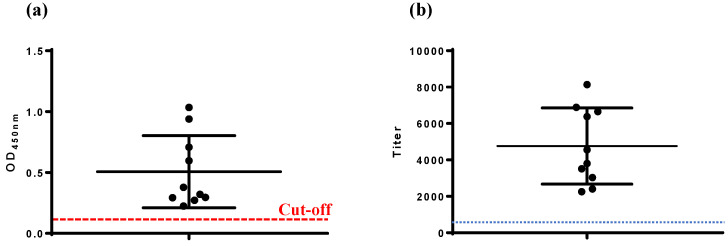
Detection of serum antibodies in chickens immunized with IBD live vaccine. (**a**) VP2-ELISA, (**b**) IBDV-ELISA. The cut-off values of VP2-ELISA (OD_450_, 0.111) and IBDV-ELISA (antibody titer, 875) are marked.

The VP2-ELISA established in this study demonstrated a 100% serum antibody positive rate (10/10) in chickens immunized with the IBD live vaccine (Gt) at 14 d.p.i. (Figure 3a). Similarly, the commercial IBDV-ELISA also reported a 100% serum antibody positive rate (10/10) (Figure 3b), consistent with the results of the VP2-ELISA.

#### 3.4.2. Detection of Serum Samples from Chickens Immunized with Avian Quadrivalent Vaccine Containing IBD VP2

**Figure 4:**
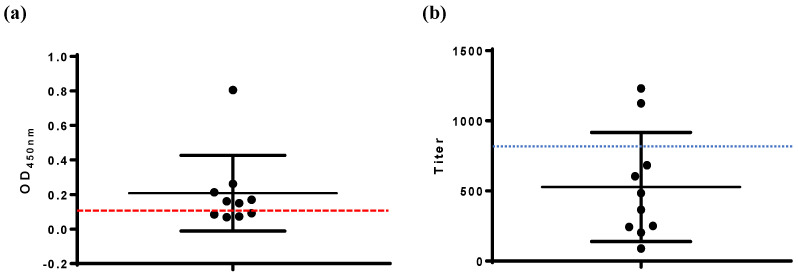
Detection of serum antibodies in chickens immunized with avian quadrivalent vaccine. (**a**) VP2-ELISA, (**b**) IBDV-ELISA. The cut-off values of VP2-ELISA (OD_450_, 0.111) and IBDV-ELISA (antibody titer, 875) are marked.

The VP2-ELISA results showed a serum antibody positive rate of 60% (6/10) for chickens immunized with the avian quadrivalent vaccine at 14 d.p.i. (Figure 4a). In contrast, the commercial IBDV-ELISA reported a serum antibody positive rate of 20% (2/10) for the same group (Figure 4b). Four serum samples yielded positive results by VP2-ELISA but negative results with the commercial IBDV-ELISA. IFA testing confirmed VP2-ELISA-positive results for all six samples, corroborating that these samples were positive for IBD antibodies.

### 3.5. Detection of Clinical Serum Samples

A total of 273 clinical chicken serum samples were tested using both VP2-ELISA and commercial IBDV-ELISA. The results showed agreement between the two methods for 231 positive and 37 negative serum samples. However, five samples were found to be positive with the commercial IBDV-ELISA but negative with the VP2-ELISA. The concordance rate between the two ELISA methods was 98.2% (268/273) (Table 2).

## 4. Discussion

After in vitro expression, the VP2 monomer of IBDV can self-assemble into VLPs under optimized conditions. VP2 in VLPs can better simulate the natural structure of VP2 [14,18]. In this study, the prokaryotic expression system of *E. coli* was utilized in order to express the VP2 protein of the predominant IBDV strain, resulting in the preparation of IBDV VLPs (SHG19-VLP), with diameters of approximately 25 nm, as observed by electron microscopy (Figure 1c). The purified IBDV-VLPs had a concentration of 500 μg/mL as determined with a BCA Protein Quantification kit, and the VLPs displayed excellent morphological integrity. These VLPs were employed as coating antigens to develop an indirect VP2-ELISA for IBDV VP2 antibodies. Compared with eukaryotic expression systems, the *E. coli* prokaryotic system for recombinant protein expression offers advantages such as lower production cost, simpler operation, and easier scalability [19]. By transforming the recombinant prokaryotic expression plasmid pCo-HHT28-SHG19VP2-466 into *E. coli* BL21 (DE3), the VP2 protein of IBDV was expressed at high levels and self-assembled into VLPs with strong reactivity. Of course, a prokaryotic expression system can sometimes lead to the formation of inclusion bodies and may result in low immunogenicity owing to potential issues with glycosylation [19].

ELISA is the preferred tool recommended by the World Organization for Animal Health (WOAH) for detecting immune responses against IBD vaccination [20]. The purity and stability of the coating antigen are critical factors in determining the quality of an ELISA [21]. Thus, the purity of the IBDV VP2 protein will directly affect the specificity and sensitivity of the indirect VP2-ELISA. To obtain high-purity VP2 protein, SHG19-VLPs were prepared using ammonium sulfate crude purification and TFF concentration technology. In specificity testing, the VP2-ELISA reacted only with IBDV-positive serum and did not exhibit cross-reactivity with sera positive for other common avian viruses, indicating excellent specificity. In sensitivity testing, the lowest serum dilution detectable by the VP2 protein, at a coating dose of 250 μg/well, was 1:6400. This sensitivity surpassed that of the commercial IBDV-ELISA coated with whole virus and IFA, demonstrating superior sensitivity. CV reflects the repeatability and stability of ELISA experiments, with smaller CV values indicating better reproducibility. A CV of less than 10% is generally considered ideal, while values below 5% are deemed excellent. The VP2-ELISA demonstrated a CV of less than 2% in both inter-batch and intra-batch repeatability tests, confirming its high stability.

The practicality of the VP2-ELISA developed in this study was evaluated by detecting antibodies in both laboratory and clinical chicken serum samples, with a commercial IBDV-ELISA coated with whole virus used as the control. Two representative IBD vaccines were selected based on their antigenic components for immunization experiments. The first vaccine, IBD live vaccine (Gt), contains the whole virus of IBDV as its antigenic component. Both the VP2-ELISA and the commercial IBDV-ELISA showed consistent results, with 100% (10/10) of the serum samples testing positive for antibodies at 14 d.p.i. (Figure 3). The second vaccine, an avian quadrivalent vaccine, is one of the IBD VP2 subunit-based vaccines in which the VP2 protein of IBDV serves as the IBD antigenic component. The VP2-ELISA detected positive serum antibodies in 60% (6/10) of the samples, while the commercial IBDV-ELISA detected only 20% (2/10) (Figure 4). Four serum samples showed inconsistent results between the two methods. Indirect immunofluorescence assay (IFA) confirmed that all four samples were positive for IBDV, consistent with the VP2-ELISA results (Figure 2). These results also indicated that compared to the IBD live vaccine (whole virus vaccine), the avian quadrivalent vaccine (containing IBDV VP2) induced relatively low levels of IBDV serum antibodies in immunized chickens. Previous clinical data have shown that when tested using ELISA coated with whole virus, viral vector vaccines, such as the recombinant MDV or the recombinant *Lactococcus lactis* expressing IBD VP2, also induced low levels of IBD antibodies [22].

These findings mentioned above suggest that for low-level serum antibody detection, the whole virus-coated IBDV antibody ELISA kits are less sensitive and prone to false negatives, consistent with the findings of other researchers [17]. However, the IBDV antibody ELISA kits currently approved for use in various countries, including China, rely on whole virus coating antigens [23], which are not optimal for evaluating the immune efficacy of IBD VP2 subunit-based vaccines. To address this clinical limitation, the VP2-ELISA developed in this study used VP2 protein as the coating antigen, offering high sensitivity. This method is suitable not only for detecting the immune efficacy of traditional whole virus vaccines but also for IBD VP2 subunit-based vaccines wherein VP2 is the primary antigenic component, presenting promising application prospects. Furthermore, to evaluate the practicality of the VP2-ELISA in clinical serum detection, 273 clinical serum samples were tested alongside the commercial IBDV-ELISA. The concordance rate between the two methods was 98.2%, demonstrating the reliability and potential utility of VP2-ELISA for clinical applications.

The development of indirect ELISA for detecting IBDV VP2 antibodies has received increasing attention [17,23]. Commercial VP2-ELISA kits, such as ID-VET products, are available in many different countries. However, similar products for the strains prevalent in China have not yet been developed. The coating antigen used in the VP2-ELISA developed in this study was a VLP prepared from the VP2 of a novel variant of IBDV, which is currently prevalent in China [2,24]. The practicality of VP2-ELISA and its comparison with other ELISA kits needs to be validated and optimized by detecting more clinical samples in the future.

## 5. Conclusions

In summary, this study developed an indirect VP2-ELISA for detecting IBDV VP2 antibodies, demonstrating excellent specificity, sensitivity, stability, and strong consistency with commercial test kits. The VP2-ELISA offers considerable advantages in immune response detection for IBD VP2 subunit-based vaccines, where VP2 serves as the antigenic component of IBDV. This method is highly suitable for evaluating the efficacy of IBD vaccines and detecting clinical serum samples.

## Figures and Tables

**Table 1:** The inter-repeatability and intra-repeatability of the assay.

Sera	Intra Batch CV%		Inter Batch CV%	
	X ± SD	CV%	X ± SD	CV%
1	1.737 ± 0.005	0.3%	1.992 ± 0.034	1.7%
2	2.032 ± 0.021	1.0%	2.045 ± 0.034	1.6%
3	1.849 ± 0.008	0.4%	2.141 ± 0.033	1.5%

Means ± standard deviations (SDs) of three replicates are shown for inter-assay and inter-assay variability.

**Table 2:** The detection of clinical samples using this VP2-ELISA and a commercial IBDV-ELISA kit.

VP2-ELISA	IBDV-ELISA		
	Positive	Negative	Total
Positive	231	0	231
Negative	5	37	42
Total	236	37	273
Coincidence	97.9%	100%	98.2%

## Data Availability

Data can be requested by writing to the author.
